# State of the JACMP: Thoughts on our transition and a hearty welcome to our new publishing partner, Wiley

**DOI:** 10.1002/acm2.12028

**Published:** 2017-01-19

**Authors:** Michael D. Mills

I want to thank our new publishing partner Wiley for a remarkably smooth changeover and for producing this first issue of the JACMP on the new publishing platform. As you explore the table of contents, I hope you will notice some new features.

The migration of the archive from the old site is still in progress, however, you will notice some improvements. We are assigning new digital object identifiers (DOI) to each of the articles from past JACMP issues to be compliant with current publishing practices. A digital object identifier (DOI) is a unique alphanumeric string assigned by a registration agency (the International DOI Foundation) to identify content and provide a persistent link to its location on the Internet. The publisher assigns a DOI when your article is published and made available electronically. The DOI is like a digital fingerprint: Each article receives a unique one at birth, and it can be used to identify the article throughout its lifespan, no matter where it goes. Developed by a group of international publishers, the DOI System provides a way to guarantee that digital copies of articles can remain accessible even if a journal changes its domain name or ceases publishing. DOIs are assigned and maintained by registration agencies such as CrossRef (http://www.crossref.org/) which provides citation‐linking services for scientific publishers.

You will notice that Wiley provides an abstract, a Full Text (HTML), a PDF version, and a Reference list for all articles. If you mouse over the title of the article, a box containing the abstract will appear. Open the reference list and you will find CrossRef links to the articles and Web of Science Times Cited numbers for the references listed. Finally, Wiley and the JACMP will post accepted articles as soon as they are available. Six times per year, Wiley will collect these articles, finalize the page numbers and publish a new issue of the JACMP. All these features will add to the value of the JACMP for the worldwide clinical physics community that we serve. Thanks to our readers, reviewers, editors, and the publication team as we finalize this transition and welcome the beginning of our future partnership with Wiley.

## Conflict of Interest

None.

